# Preferences for Communication About Prognosis Among Children With Cancer, Parents, and Oncologists

**DOI:** 10.1001/jamanetworkopen.2025.5431

**Published:** 2025-04-16

**Authors:** Caroline Christianson, Calliope Reeves, Harmony Farner, Shoshana Mehler, Tara M. Brinkman, Justin N. Baker, Pamela Hinds, Jennifer W. Mack, Erica C. Kaye

**Affiliations:** 1 Division of Pediatric Hematology/Oncology, NYU Langone Health, New York City, New York; 2 Rhodes College, Memphis, Tennessee; 3 Department of Oncology, St Jude Children’s Research Hospital, Memphis, Tennessee; 4 Department of Psychology and Biobehavioral Sciences, St Jude Children’s Research Hospital, Memphis, Tennessee; 5 Department of Epidemiology and Cancer Control, St Jude Children’s Research Hospital, Memphis, Tennessee; 6 Department of Pediatrics, Stanford University, Palo Alto, California; 7 Department of Nursing Science, Professional Practice & Quality, Children’s National Hospital, Washington, DC; 8 Department of Pediatrics, School of Medicine and Health Sciences, The George Washington University, Washington, DC; 9 Department of Pediatric Oncology, Dana-Farber Cancer Institute, Boston, Massachusetts; 10 Division of Population Sciences’ Center for Outcomes and Policy Research, Dana-Farber Cancer Institute, Boston, Massachusetts

## Abstract

**Importance:**

Pediatric oncologists infrequently ask patients and parents about their preferences for receiving information about prognosis prior to initiating difficult conversations. The willingness of patients, parents, and oncologists to engage in discussions about prognostic disclosure preferences and their perceptions of best practices in these discussions remain understudied.

**Objective:**

To explore and characterize recommendations from patients with pediatric cancer, as well as parents and oncologists, for if or how oncologists should elicit communication preferences regarding prognosis in advanced pediatric cancer.

**Design, Setting, and Participants:**

This qualitative study used responses from patients with pediatric cancer, parents, and oncologists in single interviews conducted between 2022 and 2023. Eligible patients were aged 12 years or older and recruited from an academic pediatric cancer center and 5 affiliated referring centers across 5 states. Interviews were conducted at different time points in the advancing illness course. A framework for patient-clinician communication domains was used to organize interviews and summarize data, with rapid analysis conducted to generate themes.

**Exposures:**

Poor prognosis pediatric cancer at different time points across the illness course: diagnosis, relapse or disease progression, phase 1 or 2 trial enrollment, and bereavement.

**Main Outcomes and Measure:**

Identification of themes regarding patient, parent, and oncologist preferences for eliciting and disclosing prognostic information.

**Results:**

The 85 participants surveyed included 25 patients (13 patients aged 12-14 years [52%]; 14 male [56%]; 6 Black [30%], 18 White [65%]), 40 parents (32 female [80%]; 14 Black [35%], 24 White [60%]), and 20 oncologists (14 female [70%]; 6 Asian [30%], 13 White [65%]), nearly all wanted to receive prognostic disclosure. Some patients, most parents, and most oncologists advocated for prognostic disclosure even if not requested or desired by patients or their parents, with 2 themes driving this recommendation: placing trust in medical experts and needing to stay informed. Most patients and parents, and some oncologists, emphasized the benefits of eliciting communication preferences before disclosing prognosis, generating 2 main themes: the value of individualized information and protecting mental and emotional well-being of patients and parents. Participants recommended targeted strategies to elicit prognostic communication preferences guided by 3 themes: ask questions, give options, and consider delivery or tone.

**Conclusions and Relevance:**

In this qualitative study of prognostic communication preferences, patients, parents, and oncologists recommended eliciting patient and parent preferences for prognostic disclosure in advance and provided advice for achieving this goal. Future work will integrate these recommendations in the development of targeted interventions to support individualized prognostic disclosure in advanced pediatric cancer.

## Key Points

**Question:**

Should oncologists elicit communication preferences from patients with pediatric cancer and their parents before disclosing prognosis?

**Findings:**

In this qualitative study involving 85 participants (25 patients, 40 parents, and 20 oncologists), nearly all participants advocated for individualized prognostic communication, recommending that oncologists proactively ask about communication preferences prior to sharing prognostic information.

**Meaning:**

In this study, patients, parents, and oncologists recommended preemptive elicitation of communication preferences with the goal of improving alignment of prognostic disclosure with patient and caregiver communication needs, thereby enhancing quality of care.

## Teaser

This qualitative study uses interviews of patients with pediatric cancer and their parents and oncologists to examine preferred approaches to communication about prognosis.

## Introduction

Most pediatric patients with cancer and their parents want to receive clear and frequent information about prognosis,^1,2,3,4,5,6,7^ yet a growing body of evidence demonstrates ongoing deficits in provision of timely, person-centered prognostic communication,^8,9,10^ including a mismatch between what pediatric oncologists know about prognosis and what they communicate to patients and families at the bedside.^11^ To date, efforts to improve prognostic communication have focused primarily on promoting clear and compassionate information exchange^12^; little attention has been placed on clinical, educational, and research efforts to guide clinicians in eliciting patient and parent preferences for prognostic communication downstream in the illness course to inform discussions at future time points of disease recurrence or progression.

Elicitation of prognostic communication preferences as a mechanism to inform subsequent conversations across advancing illness may seem intuitive; however, data suggest that pediatric oncologists rarely ask patients and caregivers about their preferences for receiving information about prognosis prior to initiating difficult conversations.^13^ Given recent evidence that oncologists often defer or soften dialogue about poor prognosis,^9,10,14^ it is perhaps unsurprising that oncologists may evade conversations about prognostic communication preferences as a gateway or segue into these high-stakes conversations. Reticence by pediatric oncologists to bring up topics related to prognosis during time points of disease stability^14^ also may hinder preemptive exploration of prognostic communication preferences during quiescent phases of illness prior to disease recurrence or progression.

In lieu of eliciting prognostic communication preferences, pediatric oncologists often infer what patients and parents want, need, or are ready to hear,^13^ relying on their intuition or best judgement. Despite the best of intentions, oncologists may not always guess correctly, particularly given 2024 data that patients, parents, and oncologists hold diverse, and at times conflicting, preferences for the right way to discuss prognosis.^13^ Up-front elicitation of prognostic communication preferences as standard of care has the potential to mitigate this disconnect; however, before integrating this practice routinely into bedside care, education, and research, it is imperative to explore the perspectives of patients, parents, and clinicians on their willingness to engage in discussions about prognostic disclosure preferences as well as their views on best practices for eliciting these preferences. To address these gaps, this article presents findings from interviews with children, adolescents, and young adults with poor prognosis cancer, parents of children receiving active cancer-directed therapy, bereaved parents, and oncologists to explore recommendations for eliciting prognostic communication preferences with the goal of tailoring clinical care, education, and research to improve person-centered prognostic disclosure.

## Methods

The RIGHTime (Revealing Information Genuinely and Honestly across Time) study is a multiphase, mixed-methods investigation of strategies to improve individualized prognostic communication in pediatric cancer. This article reports findings from phase 1 of the RIGHTime study, in which semi-structured interviews were conducted with patients, parents, and pediatric oncologists. This study was developed and implemented by a multidisciplinary research team (eTable 1 in Supplement 1) in partnership with bereaved parents who provided guidance on study design and materials.^12^ The study protocol was reviewed and approved by the St Jude Children’s Research Hospital institutional review board. Eligible patients and parents were approached in person during clinic visits or by telephone to conduct written informed consent as per institutional policy. Oncologists were contacted via email and completed verbal consent. We report methods and findings in accordance with the Consolidated Criteria for Reporting Qualitative Research (COREQ) reporting checklist.^15^ An overview of the study timeline is presented in eFigure 1 in Supplement 1.

### Eligibility, Recruitment, and Enrollment

Eligible patients were aged 12 to 25 years and diagnosed with cancer with estimated survival of 50% or below as determined by an oncologist. Patients were invited to participate in a single time-point interview if they were within 3 months from one of the following time points: a poor-prognosis diagnosis (cohort 1), disease relapse and/or progression (cohort 2), or enrollment on a phase 1 or 2 trial (cohort 3). Eligible parents were aged 18 years or older, caregivers of patients of any age who had cancer with a poor prognosis, and recruited from 1 of the 3 time points described previously and during bereavement 6 to 24 months after their child’s death (cohort 4). Participating patients and parents received care at the central study site at some point in the illness course, although many also received care from other institutions. Eligible clinicians were pediatric oncologists from the central study site and 5 other institutions with pediatric cancer programs, representing a total of 6 centers across 5 states. Participants were all English-speaking. Research team members (H.F., S.M., and E.C.K.) reviewed inpatient and outpatient rosters for eligible patients and parents, and reviewed online directories to identify eligible oncologists. Purposeful sampling was conducted to enroll participants with demographic diversity related to gender, age, race, ethnicity, and disease type (for patients and parents) or years in clinical practice (for oncologists).

### Interview Guide Development and Data Collection

Interview guides specific to parents, patients, and oncologists were developed by the research team, using the National Cancer Institute (NCI) domains for patient-clinician communication as a framework^16^ to ensure comprehensive generation of question prompts and probes across multifaceted aspects of communication quality. The process for interview guide development was previously published, including pilot testing of questions with oncologists and parents of children with cancer to elicit feedback, informing iterative revisions.^12^ Trained interviewers at the central study site (H.F., S.M., E.K.) conducted private, semi-structured interviews in person in the clinic or hospital setting or via telephone at the participant’s discretion, such that participants were physically situated in various geographic locations across multiple states during the interviews. Field notes were taken by interviewers, and interviews were audio-recorded, transcribed, and deidentified.

### Data Analysis

This analysis focused on 2 interview prompts with multiple corresponding probes (Box 1) aligned with the NCI core communication domains of enabling self-management (ie, patient or caregiver ability to manage aspects of their illness, including how they receive prognostic communication) and information exchange (ie, patient or caregiver preferences for discussing prognosis).^16^ A rapid qualitative approach was employed to synthesize large data for efficient intervention development and testing.^17,18^ The research team created episode summary templates to extract the raw data, using the NCI communication domains to organize the data. Analysts (H.F., S.M., E.C.K) independently reviewed transcripts, wrote memos, and pilot-tested the templates independently, inductively generating discrete concepts for each section of the episode summary. The team met regularly to review variances in generated concepts until more than 90% consensus was achieved across summaries. Analysts then completed episode summaries independently for each transcript, cross-auditing 10% of transcripts for consistency. Data from episode summaries were organized in a matrix to compare concept patterns across participants. Through iterative team discussion, high-level themes were identified and compared and contrasted across cohorts. Frequency of emerging concepts or themes were reported by quartiles: several (0% to 24%), some (25% to 49%), most (50% to 74%), or nearly all (75% to 100%). Patients were stratified by ages 12 to 15 years, 16 to 19 years, and 20 years or older to explore possible differences in preferences by age. Participants self-reported gender, race, and ethnicity during interviews; these demographics were reported descriptively to provide information on the composition of the study sample.

Box 1. **Targeted Interview Prompts and Probes**

**Patients and Parents**

Do you want to know information about prognosis? Why or why not?

- Do you think it’s useful or important to know information about prognosis?
- Does this information help you make decisions about treatment?
- Does it help you figure out next steps? Prepare for the future?
- If no: would you want to hear parts of the information? Have other family members hear it instead? Hear it at a later time?

Should a doctor ask you ahead of time about what information you want to hear?

- What’s a good way to ask this question?
- If a patient or parent does not ask directly for prognosis information, should the doctor still share it?

**Oncologists**

Do you routinely ask patients or families what information they want to hear before sharing information about prognosis?

- If so, what’s a good way to ask this question?
- If not, why not?
- If a patient or parent does not ask directly for prognosis information, should the doctor still share it?

## Results

Eighty-five semi-structured interviews were conducted with 25 patients (5 in cohort 1, 10 each in cohorts 2 and 3; 11 female [44%]; 6 Black [24%], 18 White [72%]), 40 parents (10 each in cohorts 1-4; 32 female [80%]; 14 Black [35%], 24 White [60%]), and 20 oncologists (10 at the central site, 10 from other sites; 14 female [70%]; 6 Asian [30%], 13 White [65%]) (Table 1). Median (IQR) interview duration was 25 minutes for patients (19-38 minutes), 53 minutes for parents (19-140 minutes), and 47 minutes for oncologists (26-88 minutes).

**Table 1.** Participant Demographics

Characteristics	Participant, No. (%)
**Parents (n = 40)**	
Sex	
Male	8 (20)
Female	32 (80)
Race	
African American or Black	14 (35)
Asian	1 (5)
Middle Eastern	0
White	24 (60)
2 or More races	1 (5)
Ethnicity	
Hispanic or Latino	5 (13)
Non-Hispanic	35 (87)
**Patients (n = 25)**	
Sex	
Male	14 (56)
Female	11 (44)
Race	
African American or Black	6 (24)
Asian	0
Middle Eastern	0
White	18 (72)
2 or More races	1 (4)
Ethnicity	
Hispanic or Latino	3 (12)
Non-Hispanic	22 (88)
Patient age at interview	
12-14 y	13 (52)
15-17 y	7 (28)
18-20 y	3 (12)
21-25 y	2 (8)
**Oncologist (n = 20)**	
Sex	
Female	14 (70)
Male	6 (30)
Race	
African American or Black	0
Asian	6 (30)
Middle Eastern	1 (5)
White	13 (65)
2 or More races	0
Ethnicity	
Hispanic or Latino	1 (5)
Non-Hispanic	19 (95)
Clinical practice experience	
0-5 y	5 (25)
5-10 y	6 (30)
>10 y	9 (45)

The domains used to organize the interview guide and abstract data from the interview transcripts (ie, enabling self-management and information exchange) broadly encompassed the findings generated by this targeted analysis. To minimize redundancy, results were organized not by domain and instead by participant perspectives on 4 topics of clinical relevance: (1) desire for prognostic disclosure, (2) prognostic disclosure when a patient or parent does not want the information, (3) preferences for asking about prognostic communication preferences before disclosing information, and (4) strategies for upfront elicitation of prognostic communication preferences.

Analysis of these interviews revealed substantial thematic consensus, with nearly all patients and parents wishing to hear information about prognosis and most patients, parents, and oncologists supporting elicitation of communication preferences prior to sharing information about prognosis. Furthermore, patients, parents, and oncologists offered overlapping strategies for how oncologists should approach this conversation, and the Figure synthesizes their recommendations into a simple educational framework to guide clinicians in eliciting prognostic communication preferences. Across the main themes, relatively few differences were identified between cohorts or age groups, suggesting that individualized preferences may outweigh group patterns.

**Figure.**
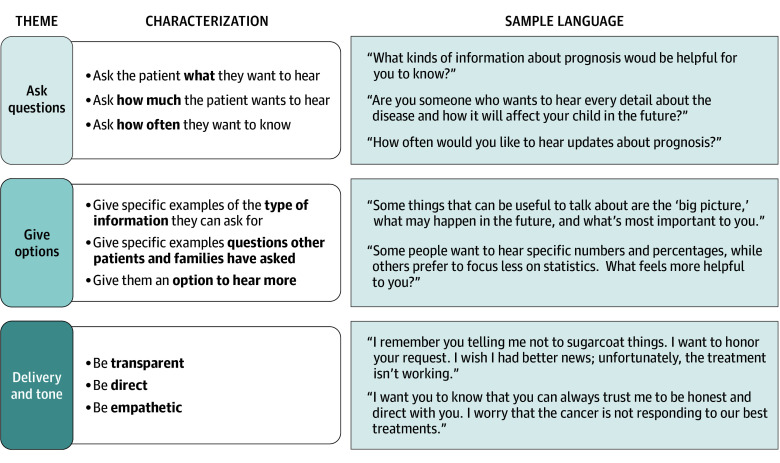
Educational Framework to Guide Clinicians in Eliciting Prognostic Communication Preferences

### Desire for Prognostic Disclosure

When patients and parents were asked, “Do you want to know information about prognosis?”, 3 main categories of responses (yes, no, unsure) were identified. The vast majority of participants across cohorts said yes, with analysis generating 3 main themes as drivers for their preference: a desire to stay informed, the importance of anticipating or preparing for the future, and their perceived value of prognostic information on decision-making. For the several participants who preferred not to hear any prognostic information, 3 core themes were identified underpinning their preference: their trust in medical experts, emphasis on a short-term mindset, and protection of mental and emotional well-being. Among the few outlier patients and parents who felt unsure if they wanted to know prognostic information, a desire to hear only specific or selective information was described along with general ambivalence about prognostic communication and self-awareness that patients and parents may not know what they want to know about prognosis. Table 2 presents quotes to illustrate different rationales across perspectives on a desire for prognostic disclosure. Granular synthesis of participant preferences regarding prognostic disclosure with additional supporting quotes are presented in eTable 2 in Supplement 1. Of note, no key differences in main themes were identified between patients and parents or between patients of different ages in this component of analysis.

**Table 2.** Prognostic Disclosure Recommendations

**Do you want to know information about prognostic disclosure?**		
**Yes, because it helps with…**	**Maybe, we have a disposition toward…**	**No, because we prefer to have…**
*Staying informed:* “It helps me at least feel not just clueless on what’s happening.” (Patient 10, 15-17 y, cohort 1)	*Selective information:* “Sometimes…some days it wasn’t so easy to listen to, after a long day.” (Patient 5, 18-20 y, cohort 2)	*Trust in medical experts:* “I fully trust that the staff here has [child’s name]’s best intentions…we are fully trusting their plan.” (Parent 23, cohort 1)
*Anticipating and preparing for the future:* “I want to be prepared to help my daughter in any way possible, so I need to know what’s going to happen.” (Parent 12, cohort 3)	*Ambivalence:* “It depends. If it’s, I don’t know, bad, good, scary, I don’t know.” (Patient 11, 12-14 y, cohort 2)	*Short-term mindset:* “Right now, we’ve just been taking it day by day, trying not to think about the future too much. Just…getting through that day because if we start thinking about the future too much, it gives us some anxiety.” (Parent 24, cohort 1)
*Decision-making:* “Well, I like to make my own decisions.” (Patient 12, 12-14 y, cohort 2)	None	*Mental and emotional well-being:* “I don’t feel like I’m in a place that I can handle hearing something that’s going to put a negative thought process going.” (Parent 8, cohort 3)
**If a patient or parent does not ask for prognosis information, should the doctor still share it?**		
**Yes, we prefer…**	**Maybe, we affirm…**	**No, we prioritize…**
*Trust in medical experts:* “When it comes to talking with parents, I feel like there’s some duty to share the full scope of the case….I can’t imagine finding out down the road that there’s some part of [child’s name]’s full picture that hasn’t been shared with me because I didn’t know to ask.” (Parent 16, cohort 2) “So for certain people that aren’t used to it…they’re not quite sure what questions to ask…so it is important to know.” (Patient 14, 12-14 y, cohort 3) “I believe that the parent and patient, for example who is a teenager, I think that they are entitled to know that things are not gonna go well….So although I don’t like to use percentages, I think it’s important to have a frank conversation of what we’re dealing with.” (Oncologist 1)	*A case-by-case approach:* “I have mixed emotions on that because I don’t know if I’d want that shared in front of [patient].” (Parent 14, cohort 3) “I believe it all depends on the situation to be quite honest with you when it comes to that. I mean, I believe it kind of depends on the situation, the age of the person.” (Patient 15, 18-20 y, cohort 3) “So parents…education level is one of the most important thing, how well informed are they? And what is their social background? What profession they are from. And if it is a single mom, or just is coming from a big supportive family? Different criteria play into the role and what kind of message am I about to give them.” (Oncologist 2)	*Mental and emotional well-being:* “I don’t feel like I’m in a place that I can handle hearing something that’s going to put a negative thought process.” (Parent 8, cohort 3) “No, because it could freak them out.” (Patient 10, 15-17 y, cohort 1) “I have one patient right now where I feel like every time I see them, I just am giving them bad news. Like, every single time and so it needs to be a balance. Where I don’t want them to feel like they’re continually like, I’m causing trauma every time I see them.” (Oncologist 3)
*Staying informed:* “I think, yes. I feel like the more you know about your child and what is going on, it will help you.” (Parent 26, cohort 4) “Probably should tell you so you don’t do something that will hurt you.” (Patient 16, 12-14 y, cohort 2) “I do tell them. I make it clear that this is the prognosis, even if they don’t ask. I think they need to know, they need to be prepared, and frankly, for to start palliative care as early as possible.” (Oncologist 4)	*Ambivalence: *“I don’t know. Not knowing it’s possible to know is hard to tell, how much I want to know.” (Parent 25, cohort 3)	*Respecting personal preferences:* “I feel like maybe they should ask the parent when’s a good time for them. If the parent doesn’t want to hear it, of course, don’t tell them because it’s just not respectful.” (Parent 24, cohort 1) “I feel like if the patient doesn’t want to know the information or didn’t ask, that the doctor should ask them first if they want to know, not just tell them.” (Patient 17, 12-14 y, cohort 1) “I will address it, if the family says to me something that sounds like an inquiry at any level. I was reading such and such newspaper and saw this number.” (Oncologist 5)
**Should a doctor ask you ahead of time about what information you want to hear?**		
**Yes, we prioritize…**	**Maybe, we affirm…**	**No, we prefer…**
*Individualized information:* “I think that’s always a good thing to ask because I might be completely different from somebody else’s parents. Somebody else might want a bunch of facts and figures and so I think it’s probably a good idea for the doctor to just go ahead and ask how.” (Parent 21, cohort 2) “The doctor should probably discuss exactly that with the patient, how much they’d want to know.” (Patient 2, 18-20 y, cohort 1)	*A case-by-case approach:* “You see patients come in with all different capacities for understanding, right? So, you don’t want to overwhelm people. You don’t want to bore people. You certainly don’t want to unnecessarily scare people, right?” (Parent 4, cohort 3)	*Trust in medical experts:* “I didn’t know which questions to ask.” (Parent 3, cohort 2) “I think I would like [the doctor] to not ask me, just say everything, because he’s the one that knows more, so I think it would be helpful for—he’s not authority, but he knows more about it.” (Patient 12, 12-14 y, cohort 2)
*Mental and emotional well-being:* “That sounds like a good way to invite the truth in….It prepares you for the magnitude of feelings you’re about to be hit with, so I think that could be helpful.” (Parent 28, cohort 1) “Probably from my perspective, yes, I guess, because for me, I guess it’s—it is still very hurtful sometimes to hear certain things.” (Patient 14, 12-14 y, cohort 3)	*Ambivalence:* “I don’t know...it’s hard for them to tell me a prognosis on this because I hate to go up and say, ‘What’s the probability he’s going to survive?’ That’s kind of a hard thing for a doctor to say, an unknown type of thing.” (Parent 25, cohort 3)	*Responsibility to share important information:* “No, I guess they know they should use like the most important about the situation at the moment and the plan for the immediate future meaning like this month, this year.” (Parent 27, cohort 3) “[Our oncologist] said she was just going to tell us the need-to-know. If we had any questions, we could ask her.” (Patient 10, 15-17 y, cohort 1)

### Providing Prognostic Disclosure in the Absence of Patient or Parent Request for Information

When asked whether the oncologist should share prognostic information regardless of whether the patient or parent wanted this information, participant responses were again categorized as yes, no, and unsure. Most parents and oncologists advocated for prognostic disclosure even if not requested or desired by patients or parents; at least some patients, representing all age groups, also endorsed this approach. Across cohorts, 2 main themes emerged to explain this preference: placing trust in medical experts and needing to stay informed. In the latter theme, both patients and parents described how prognostic disclosure helped them prepare for the future, whereas oncologists focused more on the role of prognostic information in setting expectations and decision-making. Among the few outliers who advised that oncologists should not share prognostic information if patients or parents did not request it, 2 familiar themes emerged: protecting mental and emotional well-being and respecting personal preferences. Across both themes, patients, parents, and oncologists recommended that clinicians should ask questions to elicit preferences and then tailor communication. For the small number of participants who expressed uncertainty, the main theme identified was the need for a case-by-case approach. Patients and parents more often highlighted patient age, conversation timing, and personality as variables that might influence preferences, whereas oncologists focused more on health care literacy and their own perceptions of patient and parent readiness for information to guide their approach for prognostic disclosure. Themes and supporting quotes are presented in Table 2, with additional synthesis and quotes provided in eTable 2 in Supplement 1.

### Eliciting Communication Preferences Before Prognostic Disclosure

When patients, parents, and oncologists were asked, “Should a doctor ask you ahead of time about what information you want to hear?”, we identified a similar pattern and spread for the following basic response categories: yes, no, and unsure. Most patients and parents and some oncologists emphasized the need for clinicians to ask about communication preferences before disclosing prognosis, describing 2 core reasons for this preference: the value of individualized information and protecting mental and emotional well-being of patients and parents. Less commonly, participants across each of the cohorts shared divergent perspectives on why oncologists should not elicit prognostic disclosure preferences, with 2 related themes identified as drivers for this preference: trust in medical experts to disclose necessary information and physicians’ ethical responsibility to disclose prognosis regardless of patient and parent preferences. Finally, several parents, but no patients or oncologists, expressed ambivalence, noting that prognostic information may not be conclusive and thus may not be important to some families. Identified themes were present across cohorts and ages (Table 2); possible trends with respect to different frequencies of themes described between cohorts or age groups are described in eTable 2 in Supplement 1 with additional quotes.

### Strategies for Eliciting Prognostic Disclosure Preferences

Analysis of participant recommendations for how oncologists should elicit prognostic communication preferences generated 3 broad themes: ask questions, give options, and consider delivery and tone. Most patients, parents, and oncologists advocated for creating a comfortable and open space that promoted conversation. Most patients and parents wanted oncologists to provide options with examples of different types of prognostic information that could be shared, believing that this approach might encourage discussion of prognostic preferences while simultaneously guiding families through the conversation. Some patients (aged 12 to 15 years) and several parents recommended that doctors speak to parents first to hear their preferences about sharing information with the patient, advising that the age and maturity of the patient should influence if and how prognostic communication preferences are elicited. Box 2 presents each theme in greater detail with illustrative quotes.

Box 2. **Recommended Strategies for Eliciting Prognostic Communication Preferences**

**Ask Questions**

“Start off by asking, how much of this information do you want to know? What questions do you have for me first? Let’s get those questions out of the way. Answer those. If there is more information that this doctor needs to tell you, they can ask you, do you want to know this or do you want to know that? You know, do you want to know lifespan? Do you want to know medications, things like that?” (Parent 12, cohort 3)
“Saying something like, ‘How much information do you wanna hear about how the cancer affects your future?’” (Patient 16, 12-14 y, cohort 2)
“Some families like to have numbers, some families do not, some families just want to know what the plan is....Usually, I always ask them, what do you know? What’s your understanding of the current diagnosis or where we are right now? And sometimes elements of prognosis follow into that. So then I know kind of where they are.” (Oncologist 6)

**Give Options**

“Just that first meeting like, hey, we’re going to have a lot of conversations over the next month, years. What types of information are you going to be looking for? Do you want to know every time we talk what the updated schedule is, do you want to hear and maybe kind of give options, right? Like do you want to hear the more scientific side or do you just want me to give you the high level of what’s happening?” (Parent 5, cohort 2)
“Maybe like give examples of like what things you might want to hear and like what you might not want to.” (Patient 3, 12-14 y, cohort 3)
“I always ask them, like, is this enough information? Do you need less? Do you need more? Like, what else to try and make sure that I’m with them.” (Oncologist 3)

**Delivery and Tone**

“We appreciate transparency, so like full transparency. Just tell us what’s going on.” (Parent 4, cohort 3)
“In some cases people feel like it’s not what you say it’s how you say it.” (Parent 9, cohort 3)
“Very sweet and nice. Calm talking.” (Patient 19, 15-17 y, cohort 3)
“Directly. Directly asking how much information the patient wants to know.” (Patient 4, 15-17 y, cohort 2)
“No sugarcoat.” (Patient 20, 12-14 y, cohort 3)

## Discussion

These findings demonstrate that patients and caregivers are open to discussing prognostic communication preferences, and that oncologists also recognize the potential value in this communication approach, even as they rarely engage in it. We believe that these data are important for 3 main reasons: (1) we hope that these findings may empower clinicians at the bedside to consider broaching conversations about prognostic communication preferences in advance of disease reevaluation discussions, recognizing that patients and families may welcome these conversations; (2) these findings enable us to advocate for integration of specific skills for eliciting prognostic communication preferences within communication skills training programs and curricula; and (3) these data can help researchers better partner with patients and parents to codesign interventions that promote individualized prognostic disclosure in the setting of pediatric cancer, with the potential for expansion of this paradigm to other serious illness settings.

Notably, these data affirm previous research demonstrating that most pediatric cancer patients and parents want frank prognostic disclosure,^1,2,3^ while providing additional insights into why this information matters to families. Individualized prognostic communication was championed by all participants, including oncologists; yet a 2024 study^13^ shows that pediatric oncologists rarely ask about communication preferences before sharing prognostic information, suggesting the value of teaching this practice in communication skills training (CST). Presently, few CST programs in pediatric oncology offer guidance on how to elicit prognostic disclosure preferences.^12^ Experiential learning through role play, particularly in collaboration with bereaved parent educators, may be an effective modality for incorporating this training.^19,20^

Importantly, many patients, parents, and oncologists believed that prognostic information should be shared even if families do not request it directly. On the surface, this approach may seem to contradict the goal of aligning prognostic disclosure with patient and parent preferences. However, Back et al^21^ proposes a framework to guide medical oncologists in sharing essential information while still honoring preferences: first, determine whether information is critical to share (eg, is informed decision-making contingent on prognostic understanding); second, explore preferences for prognostic communication to individualize how information is shared. We believe that targeted CST can ease this tension by teaching clinicians to elicit communication preferences and then use these preferences to guide prognostic disclosure when not directly asked.

We also advocate for awareness of outlier perspectives when designing interventions to improve prognostic communication in educational and clinical spaces. Those who preferred not to receive direct prognostic information or wanted oncologists to avoid disclosure unless directly asked emphasized a desire to protect mental and emotional well-being and to respect their oncologist’s expertise. Clinicians should anticipate these drivers and mirror language used by patients and parents as scaffolding for sensitive, individualized prognostic disclosure. eFigure 2 in Supplement 1 presents a case study for how a clinician might broach prognostic disclosure in the uniquely challenging context of a patient or parent who does not share prognostic communication preferences or request information about prognosis.

### Limitations

This study has several limitations. We used purposeful sampling to enroll a diverse cohort of patients and parents from an academic cancer center and oncologists from different institutions; however, the lived experiences of the participants in this study are not inherently representative of all pediatric cancer community members. Importantly, participants may have different communication preferences than those who declined to participate. Although we enrolled participants from minoritized racial and ethnic groups proportional to regional demographics, more than half of participants were female and White, which may influence study findings. The exclusion of participants who did not speak English limits our understanding of prognostic communication preferences across languages; future work will prioritize families who speak languages other than English. Findings were not reviewed by participants in this initial phase; however, patients, parents, and oncologists will be invited to review and interpret findings during subsequent phases of intervention codesign. Finally, this analysis focused on exploring themes unique to patient, parent, and oncologist perspectives on eliciting prognostic communication preferences; ongoing analyses stratified by illness time point will explore possible changes in preferences across advancing illness to inform further nuances in tailoring prognostic disclosure to meet individualized needs over time.

## Conclusions

In summary, patients, parents, and oncologists largely agreed on the importance of tailoring prognostic communication to the specific needs of a given patient and family. Many believed that oncologists should elicit prognostic communication preferences from patients and parents to guide the disclosure of sensitive information. Future research will focus on collaborating with pediatric cancer community members to codesign clinical interventions that support individualized prognostic disclosure in advanced pediatric cancer.

## Notes

Supplement 1. **eTable 1.** Research Team Attributes and Qualifications **eFigure 1.** Study Timeline **eTable 2.** Additional Synthesis of Participant Responses **eFigure 2.** Case Study in Disclosing Prognostic Information When Not Directly Requested

Supplement 2.
Data Sharing Statement
