# Supplementary material for: Preferences for Communication About Prognosis Among Children With Cancer, Parents, and Oncologists
Source: JAMA Netw Open. 2025 Apr 16;8(4):e255431. doi: 10.1001/jamanetworkopen.2025.5431 (PMC12004199; doi:10.1001/jamanetworkopen.2025.5431)
Supplement: Supplement 1. — eTable 1. Research Team Attributes and Qualifications eFigure 1. Study Timeline eTable 2. Additional Synthesis of Participant Responses eFigure 2. Case Study in Disclosing Prognostic Information When Not Directly Requested [file jamanetwopen-e255431-s001.pdf]

Supplemental Online Content

Christianson C, Reeves C, Farner H, et al. Preferences for communication about prognosis among children with cancer, parents, and oncologists. *JAMA Netw Open.* 2025;8(4):e255431. doi:10.1001/jamanetworkopen.2025.5431

eTable 1. Research Team Attributes and Qualifications

eFigure 1. Study Timeline

eTable 2. Additional Synthesis of Participant Responses

eFigure 2. Case Study in Disclosing Prognostic Information When Not Directly Requested

This supplemental material has been provided by the authors to give readers additional information about their work.

© 2025 Christianson C et al. *JAMA Network Open*.

eTable 1. Research Team Attributes and Qualifications

**Author**	Attributes and Qualifications
C.C.	Female pediatric hematology-oncology fellow pursuing graduate-level training in clinical research and qualitative methods.
C.R.	Female undergraduate student with training in qualitative research methodology.
H.F.	Female research associate with a Master's Degree in anthropology, formal MAXDA training, and expertise in qualitative research methodology.
S.M.	Female research associate with formal MAXQDA training and expertise in qualitative research methodology.
T.M.B.	Psychologist with PhD and clinical and research expertise in pediatric psycho-oncology supportive care, grief, and bereavement.
J.B.	Male physician-scientist with a Medical Degree, extensive clinical and research expertise related to difficult communication in oncology, and clinical training and practice in pediatric hematology-oncology and hospice and palliative medicine.
P.H.	Female nurse-scientist with PhD and extensive research expertise in stakeholder-driven participatory research, with a focus on elevating the child and parent voice.
J.M.	Female physician-scientist with a Medical Degree, a Master's in Public Health, extensive research expertise in communication science, clinical training in pediatric hematology-oncology and hospice and palliative medicine, and practice in pediatric hematology-oncology.
E.K.	Female physician-scientist with a Medical Degree, a Master's in Public Health, graduate-level training in qualitative research methodology with a focus on communication science, and clinical training and practice in pediatric hematology-oncology and hospice and palliative medicine.
All authors	*No members of the research team had a personal or professional relationship established with study participants prior to study commencement. During the informed consent process, research team members informed participants about the team's goals/reasons for conducting this research; individual interviewers otherwise did not share personal feelings or reasons for conducting the research with participants during the study process. All study team members share a collective interest in partnering with patients, parents, and clinicians to improve person-centered prognostic disclosure in pediatric cancer.*

© 2025 Christianson C et al. *JAMA Network Open*.

eFigure 1. Study Timeline

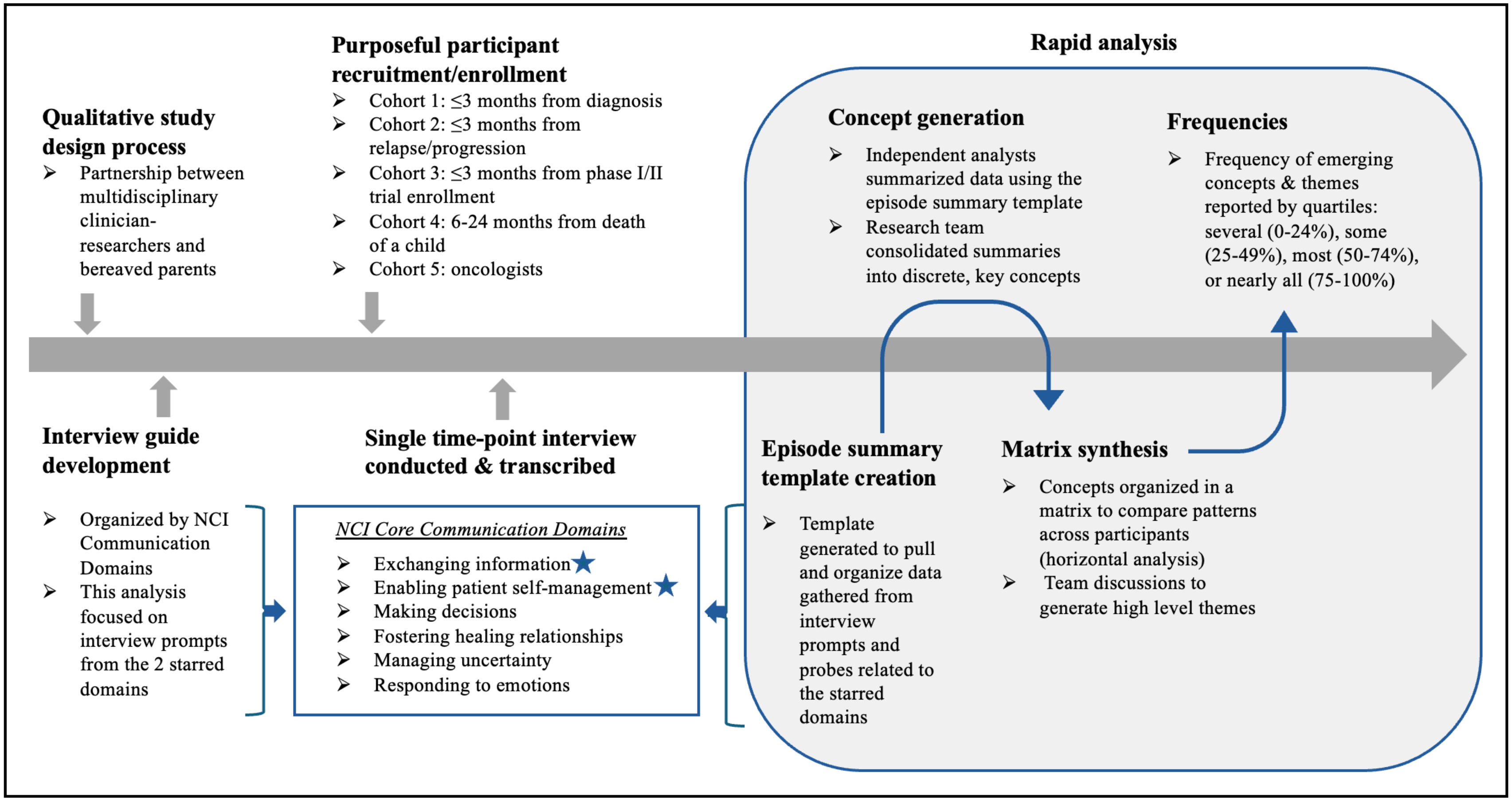

© 2025 Christianson C et al. *JAMA Network Open*.

eTable 2. Additional Synthesis of Participant Responses

Desire for Prognostic Disclosure	
Yes	Nearly all participants across cohorts expressed a desire for clear and timely prognostic disclosure, with analysis generating three core themes underpinning this preference: their desire to stay informed, the importance of anticipating/preparing for the future, and the value of prognostic information on decision-making. Most patients and parents across each age group emphasized that staying informed about prognosis was essential, and they wanted information to help them understand their disease, treatment, and the impact of cancer on their future. As one patient explained: "*Yes, because I feel like it's helps me understand everything and just like if I feel like if I don't know about something, like I'm just going to keep asking questions that my mom doesn't know the answer to.* (Patient 3, 14 years, cohort 3). Another parent corroborated this point of view, describing prognostic information as a tool that "*would make us feel like that we're not lost. That we know what's going on and even if it doesn't help you, you can't do anything, but at least you know what's happening…"* (Parent 2, cohort 1).<br><br>Patients and parents also highlighted the importance of prognostic disclosure in helping them anticipate or prepare for the future; in particular, they wanted to know what to expect from illness and treatment and believed that prognostic information alleviated anxiety caused by ambiguous or uncertain information. As one parent stated, "*I feel like I need all of the information, so that I can make a informed and educated—for me to be able to sleep at night, knowing that I put my trust in you, I need you to give me all the information and your thought process. I need you to be upfront with me, for me to be able to be comfortable and confident in our patient-doctor relationship"* (Parent 23, cohort 1). Several patients also mentioned this concept, with younger patients (12-15 years) emphasizing the importance of preparing for difficult emotions (e.g., "*I just want to understand what's happening. 'Cause I have anxiety and if I don't exactly know what's happening, my mind will just start going crazy, and I'm like, 'What does this mean? What do they mean by this?' I just want to know exactly what's happening so I can understand best and comprehend best what's happening"* (Patient 6, 15 years, cohort 2). Older patients (≥16 years) more generally described their desire to anticipate outcomes across illness ("*Yep. 'Cause, if I don't know, my mind will immediately go to the worst possible scenario. Also, knowing exactly what it is helps me be like, okay, so this is what we're going to do to not have that happen"* (Patient 4, 17 years, cohort 2).<br><br>Additionally, some parents as well as patients in each age group felt that prognostic communication facilitated goal-setting, treatment decisions, and planning around work and home life. As one parent explained, "*I like to have as much information as I can up front. I like to say we make personal decisions, family decisions based off of that information" (Parent 38, cohort 3).* Another parent further clarified, *"It's helpful because you have to make real time decisions as to what you're going to do…You know, what kind of timeline are we looking at with them not having an immune system, how can you manage them being immunocompromised…you have to make real time decisions, you know?"* (Parent 20, cohort 2).

© 2025 Christianson C et al. *JAMA Network Open*.

No	Several participants preferred not to hear any prognostic information, referencing three main reasons for this preference: their trust in medical experts, emphasis on a short-term mindset, and protection of mental and emotional wellbeing. A few patients and parents expressed their belief that doctors are better equipped to understand and interpret prognostic information, and they felt more comfortable deferring to the doctor to use this information to inform next steps. As one patient described, "*I'd probably go with the treatment that the doctor is most confident in, but they only ever offered one treatment, and I assumed they knew what they're doing. It seems like they do"* (Patient 2, 18 years, cohort 1). Similarly, another parent stated, "*We pretty much just trust her care team and what they have for her, and we just go along with it, and they tell us what can come along the way with that. We trust the care team over here"* (Parent 24, cohort 1).<br><br>A few patients and parents preferred to take a day-to-day approach or focus on immediate tasks instead of talking about the big picture; within this small group, patients described how a short-term mindset is pragmatic, whereas parents focused more on how this mindset helps mitigate emotional turmoil and preserve their mental health. As one patient discussed, "*I think they should just tell me the information that I need to know before things happen"* (Patient 7, 14 years, cohort 1). A parent shared a similar perspective: "*Well, yes, if it's needed from our moment. He's really good right now…if I don't need to know it because he's worrying us more…in the middle, I don't need a lot of information"* (Parent 27, cohort 3). Another parent also underscored the utility of focusing on the here-and-now, stating, "*And to make it better, short term. Obviously, long term is the goal but sometimes like knowing a lot what could it happen makes you think of that scenario and that's not happening"* (Parent 27, cohort 3).
Unsure	A few outlier patients and parents described feeling ambivalent about their desire to know prognostic information. In explanation, they expressed wanting to hear only specific information that they or their medical team believed to be immediately relevant to a given context. As one patient reported, "[I] *feel like yes and no. I feel like it gets to a certain point when it's like, okay, I don't need to know everything. Only [tell me what] I need to worry about"* (Patient 24, 12 years, cohort 3). Similarly, one parent verbalized, "*I got to rely on what the doctors tell me and I trust them in the sense that they will let me know when there's something that I need to know, and if there's something to worry about that they will tell me, but I can't, I just can't go down that road that's a very downward spiral for me" (Parent 8, cohort 3).*<br><br>Occasionally, patients and parents described a phenomenon in which they may not know what they want to know about prognosis. One patient explained, "Y*eah, it's a conversation that should probably be offered. But at the same time, especially if this is your first go around cancer or if this is just now getting diagnosed in your child, I don't think you would know really what's what, so at that it's like, okay, well, what information can you give me"* (Patient 1, 24 years, cohort 3). A few parents echoed this perspective: "*And it's sometimes it's not one of those things where we don't feel comfortable asking, we would ask, we just don't know to ask, you know what I mean? We don't know the questions to ask"* (Parent 5, cohort 2).

© 2025 Christianson C et al. *JAMA Network Open*.

Providing prognostic disclosure in the absence of patient/parent request for information	
Yes	The majority of parents and oncologists highlighted the need for clinicians to provide prognostic disclosure, even if the information was not requested or desired by patients and/or parents. As one parent said, "*I want to know. So even if we don't ask, we need to know* (Parent 22, cohort 4). An oncologist affirmed this perspective, stating, "*I think letting them know that it is poor prognosis upfront is important"* (Oncologist 7). Patients across all age groups also endorsed this approach, although the frequency of this response was relatively lower in the patient cohort compared to parent and oncologist cohorts: "*Probably should tell you, so if—you don't do something that will hurt you"* (Patient 16, 12 years, cohort 2).<br><br>Across participant cohorts, two main themes were identified: placing trust in medical experts and needing to stay informed. Parents, more than patients, described lacking confidence in their ability to ask the right questions, resulting in a preference to defer to the oncologist to decide what information they should hear. One parent shared, "*I feel like there's some duty [of the oncologist] to share the full scope of the case…I can't imagine finding out down the road that there's some part of [child's name]'s full picture that hasn't been shared with me because I didn't know to ask"* (Parent 16, cohort 2). Several patients (aged 12-15 years) highlighted trust in medical experts, although older patients did not mention this theme. As one patient described "*For me, personally, I think I would like [the oncologist] to not ask me. Just say everything, because he's the one that knows more, so I think it would be helpful"* (Patient 12, 14 years, cohort 2). Oncologists highlighted their professional responsibility to share prognosis; however, most oncologists preferred to keep prognostic disclosure general and advised withholding numbers or percentages unless directly asked for this information. One oncologist explained, "*I believe that the parent and patient, I think that they are entitled to know that things are not going to go well, although I don't like to use percentages, I think it's important to have a frank conversation of what we're dealing with. And they can stop me anytime they want…But I do believe that the child and the parents deserve to know that things are not going to go very well"* (Oncologist 1).<br><br>Several patients, some parents, and some oncologists believed that being updated/informed should supersede personal preferences because prognostic understanding is essential for high-quality care. One patient stated, "*If it's really important, then I think the doctor should [disclose prognosis]…a result of a big test or something like that"* (Patient 22, 14 years, cohort 2). Parents often agreed, with one explaining, "*Parents may not like [the prognosis], but I mean, they need to know this"* (Parent 29, cohort 4). Multiple oncologists echoed this perspective: "*I do tell them. I make it clear that this is the prognosis, even if they don't ask. I think they need to know, they need to be prepared, and frankly, for to start palliative care as early as possible"* (Oncologist 4).<br><br>Both patients and parents described how prognostic disclosure helped them prepare for the future, whereas oncologists focused more on the role of prognostic information in setting expectations and decision-making. As one patient said, "*I think [knowing prognosis] helps you. Well, first of all, you can know the results of your test and the treatment you did prior. Also, it can guide you in what's coming, so is it more treatment, is it going home. If you go home, maybe you have to do different things because of*

© 2025 Christianson C et al. *JAMA Network Open*.

	that result. I think it's important to know that, for sure" (Patient 12, 14 years, cohort 2). Another parent reflected on how hearing about prognosis earlier could have been helping, stating, *"It would have given us a better grasp on how to prepare for our family right... If we have other children at home, how are we going to plan and care for them? If we have jobs at home, how do we rotate between parents and jobs and children…?"* (Parent 14, cohort 3). Similarly, an oncologist shared, "*Yeah… I think it's important to address… I do need to make sure that they understand where things are at. So that together, we can make the best decisions for their child and their family. And so even though those conversations are hard, they are necessary"* (Oncologist 3).
No	Among the few outliers who advised that oncologists should not share prognostic information if patients/parents did not request it, two familiar themes emerged: protecting mental and emotional wellbeing and respecting personal preferences. A few patients, parents, and oncologists cited undue stress secondary to unwanted prognostic information; within this small group, patients, more than parents, believed that their preferences should outweigh the doctor's responsibility to disclose prognosis. One patient expressed, "*I believe the doctor should do this – if the mama wants to know, then let the mama know. If they don't, then respect them, you don't tell them"* (Patient 15, 20 years, cohort 3). Another patient offered an additional rationale, saying, "*Is it worth stressing over? Is it worth if it's already going to – I don't want to be harsh, but if your chances ain't good, is it best to just not tell the person…if a woman's 80-some years old and she's got cancer…[if] there's no cure, is it really worth telling her, or is it making her stress…?"* (Patient 15, 20 years, cohort 3). In this context, patients, parents, and oncologists recommended that clinicians should ask questions to elicit preferences and then tailor communication accordingly in response to information shared with patients and parents regarding their individualized preferences. One patient recommended the oncologist, "*... give examples of like what things you might want to hear and like what you might not want to"* (Patient 3, 14 years, cohort 3). Similarly, a parent suggested, "M*aybe just ask, like, 'Are you comfortable with us sharing this?' You know, and just figuring out that I feel those comfort levels"* (Parent 31, cohort 4).
Unsure	In the small group of participants who expressed ambivalence, similar themes arose: the value of a case-by-case approach and ambivalence to knowing prognostic information. Several patients and parents and some oncologists emphasized that what works for one family may not work for another. Specifically, patients and parents highlighted patient age, conversation timing, and personality as variables that might influence preferences. Oncologists focused more on healthcare literacy and their intuition about patient/parent readiness and need for information to inform their approach for prognostic disclosure if not directly asked. As one patient explained, "*I believe it kind of depends on the situation, the age of the person*" (Patient 15, 20 years, cohort 3). A parent reflected, "*I don't know if I'd want [prognosis] shared in front of [child's name]. Like is there an opportunity that you could step outside in the hallway with the parents and share that or have a one-on-one with parents aside from the patient and let the parents decide if that should be shared or not?"* (Parent 14, cohort 3). An oncologist felt that asking for preferences was unnecessary, stating, "*In most other cases, I've really not needed to ask that question. It's mostly been, [what] has worked with them over several weeks to several months – I figured out what [they wanted to know]"* (Oncologist 8).

© 2025 Christianson C et al. *JAMA Network Open*.

Eliciting communication preferences before prognostic disclosure	
Yes	The majority of patients and parents highlighted the value of oncologists eliciting communication preferences prior to disclosing prognosis; oncologists also emphasized this point, although less frequently than patients and parents. Across cohorts, the same two main themes were generated to explain this preference: the importance of individualized information and a need to protect the mental and emotional well-being of patients and parents. One patient explained, "*I think they should ask the patient what they want to know and hit those subjects then explain the rest"* (Patient 9,16 years, cohort 2). A parent commented, "*I think that every parent and every family is going to be a little bit different, so it's probably a good idea for the doctor to just kind of ask what they like or what they want"* (Parent 21, cohort 2).<br><br>Participants from all cohorts emphasized that prognostic disclosure can be overwhelming and anxiety-provoking, and clinicians should accommodate communication preferences with careful questioning to ensure that their communication aligns with the family's needs and goals. As one patient explained, "*…it is still very hurtful sometimes to hear certain things. So, like, maybe [some] that are just not tough enough to hear…it's important to maybe ask them [their preferences] before [disclosing prognosis], just in case"* (Patient 14, 13 years, cohort 3). A parent agreed, explaining, "*It really depends…I think the doctor should ask, do they want to know the full details or do they want him to partially tell them?"* (Parent 36, cohort 2). One oncologist affirmed this approach, sharing, "*As we go through, then I usually have had other conversations with families in terms of trying to learn how they might like to have news delivered. So those conversations might not necessarily happen at the time that I know that we're going to be talking about prognosis, but they might happen before if I know we're getting ready for a disease evaluation"* (Oncologist 3).<br><br>We identified a possible nuanced difference between age groups within the theme of mental and emotional wellbeing, with patients aged 12-15 raising this topic more often than patients of other ages. Otherwise, no notable differences were identified between cohorts or age groups. As one representative patient within this age group expressed, "*Yeah. I think [the oncologist] should ask [about prognostic communication preferences]. They should ask and see if the kid wants to know. Yeah. I don't think you should tell a kid right out of the gate that kind of information 'cause it's irrelevant. It really is. I mean, you're going to have to take the same amount of treatment and you're going to have to take the same amount of stuff. [Prognosis] is irrelevant. That is just fear"* (Patient 10, 15 years, cohort 1).
No	Less commonly, participants across each of the cohorts shared perspectives that diverged from the majority, describing their preference for why clinicians may not want or need to elicit prognostic disclosure preferences from patients or parents prior to sharing information about prognosis. Analysis generated two main themes underpinning this perspective: trust in medical experts to disclose necessary information and physicians' ethical responsibility to disclose prognosis regardless of patient/parent preferences. As one patient stated, "*I'd probably go with the treatment that the doctor is most confident in, but they only ever offered one treatment, and I assumed they knew what they're doing"* (Patient 2, 18 years, cohort 1). Similarly, a parent shared, "*I*

© 2025 Christianson C et al. *JAMA Network Open*.

	don't even necessarily think it's fair to ask [patients/parents] to tell you, 'Hey, what would you like me to tell you or what would you like to hear ahead of time'…I think it just has to be a part of their regular bedside manner, and they say…this is the case, these are the facts, but I can humanize it in a way to deliver it…with the most amount of compassion that I can give" (Parent 20, cohort 2). An oncologist underlined the doctor's ethical perspective to disclose prognosis regardless of patient/parent preferences, saying: "*…If somebody tells me, 'don't tell me [about prognosis]…I take a step back, but I don't listen to them. I still have to tell them. Sometimes I feel it is my job to tell them the real thing"* (Oncologist 2).<br><br>Several patients, some parents, and some oncologists felt that patients and parents should trust the doctor to tell them the 'right' prognostic information, noting that a doctor's skills and personal communication style should be respected. As one parent said, "*She doesn't ask us, but in the way that she says it, it's never anything that causes us to be anxious or any of that. She just throws things in there while she's talking…[child's name] may experience this from this chemo or that from that chemo, and then, she'll throw in what could happen, but she does it in a very gentle way"* (Parent 24, cohort 1). An oncologist reflected on their approach, describing how *"If it was an acute situation that I feel medically I have to communicate [prognosis] to at least adults, I go ahead. Others, I think there are probably are times I imply [prognosis] in how I talk…I don't really probably come out and ask, do you want to know this information? I feel like most parents ask me or ask questions leading to that"* (Oncologist 9). A few patients and parents also highlighted doctors' intrinsic ethical, moral, and professional responsibility to provide clinically relevant prognostic information, irrespective of patient/parent preferences. As one patient stated, "*If it's really important, then I think the doctor should [say it]"* (Patient 22, 14 years, cohort 2). A parent similarly shared, "*I feel like, whether I wanted it or not, I feel like my doctor should give [prognostic information] to me. I feel like, if you know something about my child, then I feel not entitled, but feel like you're obligated to – you should be obligated to tell me. It's my child. I should know what's going on"* (Parent 23, cohort 1).<br><br>Some patients, parents, and oncologists emphasized that elicitation of prognosis preferences may depend on factors pertaining to the illness trajectory, personalities of key actors, and resilience and psychological wellbeing of the patient/parent. One patient verbalized, "*I feel like that's kind of a hard question to ask, because it's – yeah, it's a conversation that should probably be offered. But at the same time, especially if this is your first go around cancer or if this is just now getting diagnosed in your child, I don't think you would know really what's at, so at that it's like, okay, well, what information can you give me. And then once I know that, then I'll be like, okay"* (Patient 1, 24 years, cohort 3). Parents likewise affirmed the importance of individualizing asking about prognostic communication preferences, with one parent stating, *"It kind of depends on where you are at that time"* (Parent 11, cohort 4). Oncologists focused on additional factors including patient age and trusting the doctor's intuition. One oncologist described their process of inferring what a patient/parent wanted to hear, stating, "*I try to set the stage when I first meet a family. Like my approach in general is like, if I'm worried about something, I'll let you know. Like a lot of what you're about to go through is going to be new to you and scary and different, and a lot of it will be very typical and standard to us. So if you're worried, let us know. I will also let you know if something's going on that's different and or more*

© 2025 Christianson C et al. *JAMA Network Open*.

	worrisome even to your team…I set the stage with that and most of the time I can get a feel from a family, like a lot of them say 'thank you, yes we want to know everything.' And there's other ones that would be like 'ok, we trust you,' and I get a sense from that point, but I don't directly ask them" (Oncologist 10).
Unsure	Several parents expressed feeling uncertain about the importance of eliciting prognostic communication preferences before disclosing information related to prognosis. Of note, no patients or oncologists verbalized ambivalence on this topic. Of the parents who were unsure, most referenced the fact that prognostic information is rarely certain or conclusive, suggesting that this lack of information surety lessens the perceived importance of communicating about prognosis in advance with patients and families. As one parent stated, "I *don't know. Not knowing it's possible to know is hard to tell, how much I want to know...it's hard for them to tell me a prognosis on this because I hate to go up and say, 'what's the probability he's going to survive?' that's kind of a hard thing for a doctor to say, an unknown type of thing"* (Parent 25, cohort 3).

© 2025 Christianson C et al. *JAMA Network Open*.

eFigure 2. Case Study in Disclosing Prognostic Information When Not Directly Requested

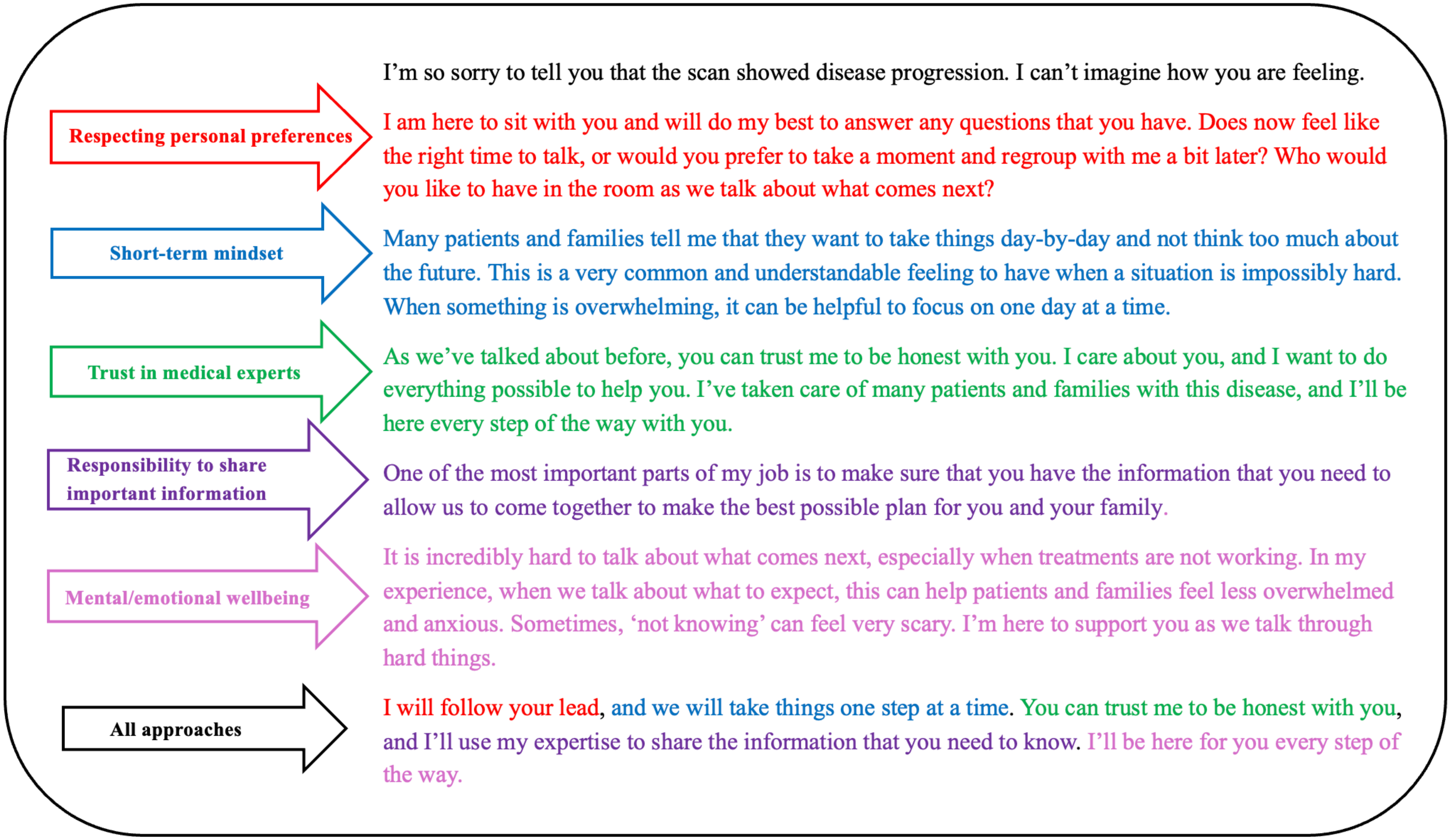

Legend. For the few patients and parents who did not want the oncologist to share information about prognosis unless they explicitly asked for it, five several key themes were identified underpinning these preferences. This figure presents example language for navigating sensitive, individualized prognostic disclosure that acknowledges and honors these preferences.

© 2025 Christianson C et al. *JAMA Network Open*.
