# Supplementary material for: Preferences for Communication About Prognosis Among Children With Cancer, Parents, and Oncologists
Source: JAMA Netw Open. 2025 Apr 16;8(4):e255431. doi: 10.1001/jamanetworkopen.2025.5431 (PMC12004199; doi:10.1001/jamanetworkopen.2025.5431)
Supplement: Supplement 2. — Data Sharing Statement [file jamanetwopen-e255431-s002.pdf]

Data Sharing Statement

Christianson. Preferences for Communication About Prognosis Among Children With Cancer, Parents, and Oncologists. *JAMA Netw Open*. Published April 16, 2025. doi:10.1001/jamanetworkopen.2025.5431

Data
Data available: Yes
Data types: Deidentified participant data
How to access data: Please reach out to the RIGHTime study principal investigator Dr. Erica Kaye to request data sharing: erica.kaye@stjude.org
When available: With publication

Supporting Documents
Document types: None

Additional Information
Who can access the data: In the context of the rarity of advancing pediatric cancer and the relatively small sample sizes intrinsic to qualitative research, a small risk exists for participant identification even following rigorous de-identification procedures. Given this risk, our research team does not share entire raw data sets upfront to all-comers. We are enthusiastic about sharing de-identified data on a case-by-case basis to researchers under a data-sharing agreement in the setting of an IRB approved research protocol and explicit assurance that data will be reviewed and analyzed exclusively for research purposes without identification of individual participants.
Types of analyses: Qualitative analyses for any purpose
Mechanisms of data availability: We are enthusiastic about sharing de-identified data on a case-by-case basis to researchers under a data-sharing agreement in the setting of an IRB approved research protocol and explicit assurance that data will be reviewed and analyzed exclusively for research purposes without identification of individual participants.
Any additional restrictions: n/a
